# Interplay of endonucleolytic and exonucleolytic processing in the 3′-end formation of a mitochondrial *nad2* RNA precursor in Arabidopsis

**DOI:** 10.1093/nar/gkad493

**Published:** 2023-06-09

**Authors:** Chuande Wang, Martine Quadrado, Hakim Mireau

**Affiliations:** Université Paris-Saclay, INRAE, AgroParisTech, Institut Jean-Pierre Bourgin (IJPB), 78000 Versailles, France; Université Paris-Saclay, INRAE, AgroParisTech, Institut Jean-Pierre Bourgin (IJPB), 78000 Versailles, France; Université Paris-Saclay, INRAE, AgroParisTech, Institut Jean-Pierre Bourgin (IJPB), 78000 Versailles, France

## Abstract

Initiation and termination of plant mitochondrial transcription are poorly controlled steps. Precursor transcripts are thus often longer than necessary, and 3′-end processing as well as control of RNA stability are essential to produce mature mRNAs in plant mitochondria. Plant mitochondrial 3′ ends are determined by 3′-to-5′ exonucleolytic trimming until the progression of mitochondrial exonucleases along transcripts is stopped by stable RNA structures or RNA binding proteins. In this analysis, we investigated the function of the *endonucleolytic mitochondrial stability factor 1* (EMS1) pentatricopeptide repeat (PPR) protein and showed that it is essential for the production and the stabilization of the mature form of the *nad2* exons 1–2 precursor transcript, whose 3′ end corresponds to the 5′ half of the *nad2 trans*-intron 2. The accumulation of an extended rather than a truncated form of this transcript in *ems1* mutant plants suggests that the role of EMS1 in 3′ end formation is not strictly limited to blocking the passage of 3′-5′ exonucleolytic activity, but that 3′ end formation of the *nad2* exons 1–2 transcript involves an EMS1-dependent endonucleolytic cleavage. This study demonstrates that the formation of the 3′ end of mitochondrial transcripts may involve an interplay of endonucleolytic and exonucleolytic processing mediated by PPR proteins.

## Graphical Abstract

**Graphical Abstract:**
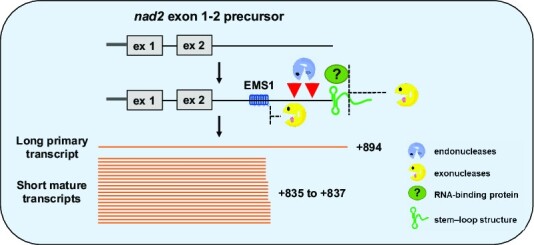

## INTRODUCTION

The mitochondria are the powerhouses of eukaryotic cells, and they contain a genome that is derived from their bacterial ancestor. The expression of the few genes present in mitochondrial genomes, which number about 50 in most organisms, requires the activity of a complete gene expression system in the organelle. Given the very limited coding capacity of mitochondrial genomes, this gene expression machinery requires importing a large number of nuclear-encoded mitochondria-targeted proteins. Many of these factors correspond to genes from the endosymbiont that have been transferred into the nucleus and whose protein products are transported from the cytosol into the organelle. Other mitochondrial factors are of nuclear-origin and have been selected throughout the evolution to assist mitochondrial gene expression. Mitochondrial gene expression thus involves a peculiar genetic organization consisting in expressing a highly degenerated bacterial scaffold (the mt genome) through the use of eukaryote-derived functions. Such an intricate genetic organization involving two physically separate genetic compartments that obey to different evolutionary trends has shaped mitochondrial gene expression processes throughout evolution. In flowering plants, the fact that mitochondrial genomes mostly evolve by genome rearrangements, resulting in generally poorly conserved gene 5′ and 3′ untranslated regions (UTR) even at the within-species level, has played a role in the shaping of mitochondrial gene expression processes. Additionally, plant mitochondrial transcription appears to be a relaxed process exhibiting hardly any control or modulation both in its initiation and termination (1,2). Thereby, precursor mRNAs in plant mitochondria are often much longer than needed and posttranscriptional events involving 5′ and 3′ processing as well as control of RNA stability are essential for proper gene expression in plant mitochondria. Several proteins belonging to the pentatricopeptide repeat (PPR) family have been shown to play roles in mature mRNA end formation in plant mitochondria (3–5). Unlike in plastids, where most genes are contained in long polycistronic transcripts (6) which requires numerous 5′ and 3′ RNA processing through both endonucleolytic and exonucleolytic cleavages to produce monocistronic mRNAs (7), mitochondrial transcripts in plants are most often produced as stand-alone transcripts. Lack of 5′-to-3′ exoribonuclease activity in plant mitochondria causes 5′ ends of mRNAs to correspond either to transcription start sites or to 5′-processed transcripts produced by endonucleolytic processing (4,8). Conversely, plant mitochondrial 3′ ends are generated by 3′-to-5′ exonucleolytic trimming of primary transcripts. In such a model, 3′-to-5′ exonucleases like the polynucleotide phosphorylase (PNPase) digest mRNAs from their primary 3′ ends until their progression is stopped either by a stable RNA secondary structure or a stabilizing protein like a PPR protein (9). Four of such PPR proteins called Mitochondrial Stability Factor 1, 2, 3 and 4 (MTSF1, 2, 3 and 4) were found to both set the 3′ end and stabilize specific mature or precursor mitochondrial transcripts (5,10–12). In the present analysis, we characterized a new RNA stabilizing mitochondrial transfactor in *Arabidopsis thaliana* that we named Endonucleolytic Mitochondrial Stability factor 1 (EMS1). We show that this PPR protein is essential for the stability of a *nad2* precursor transcript and that unlike in other mutant stability factors longer and not shorter precursor transcripts accumulate in its absence, strongly suggesting that EMS1 recruits an endonuclease for 3′ end processing of this *nad2* pre-mRNA, thereby showing that endoribonucleolytic cleavage also plays a role in 3′ end formation in plant mitochondria.

## MATERIALS AND METHODS

### Primers

Oligonucleotides used in this study are listed in Supplementary Table S1.

### Plant material and growth conditions

Arabidopsis (*Arabidopsis thaliana*) Col-0 plants were obtained from the Versailles Arabidopsis Stock Center. The N644391 (*ems1*) Arabidopsis mutant line was acquired from the European Arabidopsis Stock Centre (http://arabidopsis.info/). Homozygous *ems1* mutants were genotyped by PCR using the primers listed in Supplementary Table S1 and the insertion site was confirmed by sequencing. Plants were grown on soil in a greenhouse under long-day conditions (16 h of light and 8 h of dark).

### Functional complementation of *ems1* mutants

For mutant complementation test, the full-length EMS1 coding sequence without its stop codon was amplified by PCR, cloned into the pDONR207 vector using the Gateway BP reaction (Invitrogen), and subsequently transferred into the pGWB5 binary vector (13). The resulting construct (35S::EMS1::GFP) was transformed into *Agrobacterium tumefaciens* C58C51 and introduced into heterozygous *ems1* plants by the floral dip method (14). Functionally complemented homozygous mutant plants were identified among the obtained transgenic plants by PCR analysis.

### Subcellular localization

The roots of functionally complemented homozygous mutant plants (see above for details) were used for subcellular localization analysis. Prior to observation, roots were soaked in 0.1 μM Mitotracker™ Red (Invitrogen) to label mitochondria. The GFP fluorescence was visualized in the transgenic cell lines by Leica TCS SP5 confocal microscopy with a 40 × 1.25 numerical aperture oil objective. The filter set had an excitation wavelength/spectral detection bandwidth of 488 nm/500 to 530 nm for GFP and 561 nm/580 to 625 nm for Mitotracker™ Red.

### RNA extraction, reverse-transcription quantitative PCR and RNA gel blot

Total RNA was isolated from 8-week-old flower buds using TRIzol reagent (Life Technologies) according to the manufacturer's instructions. RNA was treated with DNase Max (QIAGEN) when RNAs were used in reverse transcription (RT)-PCR or quantitative RT-PCR (qRT-PCR) assays. Mitochondrial mRNA abundances were measured by qRT-PCR. They were calculated using the comparative ΔΔCt method after normalization to the nuclear 18S ribosomal RNA as previously described in (15). Two biological and three technical repeats were performed for these analyses.

For RNA gel blotting, 10 μg of total RNA was electrophoretically separated in formaldehyde-containing (1.5% [w/v]) agarose gels and transferred onto nylon membranes (Genescreen) as described previously (10). Hybridization probes were generated by PCR amplification using gene-specific primers (listed in Supplementary Table S1) and radiolabeled using the Prime A Gene labeling kit (Promega) according to the manufacturer's recommendations.

### Circular RT-PCR

Five μg of total RNA were circularized with 40 U of T4 RNA ligase (New England Biolabs), following the manufacturer's instructions. Circularized RNA was purified using the RNA Clean & Concentrator Kits (Zymo Research®, California, USA). The first strand complementary DNA (cDNA) synthesis was done for 3 h at 40°C using 400 U of M-MLV reverse transcriptase (Fermentas), 8 mM of random hexamers (Eurofins), 1× M-MLV buffer, 0.5 mM dNTPs and 40 U of Riboblock RNase inhibitor (Fermentas). The obtained cDNA was diluted four times, and 5 μl of the obtained cDNA solution was used for PCR amplification with divergent primers. The primers used for mapping precursor transcripts are listed in Supplementary Table S1. Amplified PCR products were gel purified, cloned into pCR2.1^®^-TOPO^®^ TA vector (ThermoFisher Scientific) and inserts of independent recombinant plasmids were sequenced after *E. coli* transformation.

### RNA immunoprecipitation assays

RNA immunoprecipitation (RIP) experiments were performed using the μMACS GFP-Tagged Protein Isolation Kit (Miltenyi Biotec) according to the manufacturer's instructions, with minor modifications. Succinctly, Arabidopsis cells expressing the 35S::EMS1::GFP translational fusion were collected from a 3-day-old culture and ground into a fine powder in liquid nitrogen. Samples were homogenized in RIP lysis buffer (20 mM HEPES–KOH, pH 7.6, 100 mM KCl, 20 mM MgCl_2_, 1 mM DTT, 1% (v/v) Triton X-100, 1× of complete EDTA-free protease inhibitor (Roche)) for 30 min at 4°C with slow rotation (10 rpm). The lysates were clarified by ultracentrifugation at 100 000 *g* for 20 min at 4°C, and 7 mg of proteins of the supernatant were incubated with 50 μl of anti-GFP magnetic beads (Miltenyi Biotec) for 1 h at 4°C with rotation (10 rpm). After four washes with 200 μl RIP of washing buffer (lysis buffer containing 0.1% Triton X-100) and one wash with 100 μl of washing buffer 2 from the kit, RNAs were extracted from the beads using TRI reagent (Life Technologies), precipitated with ethanol, and then used for RT-qPCR analysis. Prior to retrotranscription, immunoprecipitated RNAs were treated with DNase I and purified using the RNA Clean & Concentrator Kits (Zymo Research^®^, California, USA). Two independent immunoprecipitations and three cDNA syntheses prepared from each immunoprecipitation were used in the analysis and a RIP experiment with untransformed PSB-D cells was used as a negative control.

### Blue native gel and in-gel activity assays

Crude organelle extracts enriched in mitochondria were prepared as previously described (11) using eight-week-old flower buds of *ems1* mutant and Col-0 plants. One hundred micrograms of total proteins from purified organelles were loaded and separated on 4–16% (w/v) polyacrylamide NativePAGE™ Bis/Tris gels (Invitrogen). After electrophoresis, BN-PAGE gels were stained with Coomassie Blue or in buffers revealing the activities of mitochondrial respiratory complexes I and IV as previously described (16). Once sufficient coloration was obtained, gels were soaked in a fixing solution containing 30% (v/v) methanol and 10% (v/v) acetic acid to stop the reactions.

### Immunoblot analysis

BN-PAGE gels were transferred to 0.45 μm polyvinylidene difluoride (PVDF) membranes under liquid conditions in 50 mM Bis/Tris and 50 mM Tricine at 20 V overnight at 4°C. Total proteins were extracted from the crude membrane in cold lysis buffer (50 mM Tris–HCl, pH 7.5, 100 mM NaCl, 5 mM EDTA, 1% (v/v) NP-40, 0.5% (w/v) sodium deoxycholate, 0.1% (v/v) SDS, 1X of complete EDTA-free protease inhibitor (Roche)). Protein concentrations were determined with the Bradford method (Bio-Rad). Approximately 30 μg of total protein were resolved on SDS-PAGE and then transferred onto PVDF membrane under semi-dry conditions (Bio-Rad). Membranes were incubated with specific primary antibodies (Supplementary Table S2) overnight at 4°C. Hybridization signals were revealed using enhanced chemiluminescence reagents (Western Lightning Plus ECL, Perkin Elmer).

## RESULTS

### The arabidopsis *ems1* mutant displays a retarded growth phenotype

**Figure 1.**
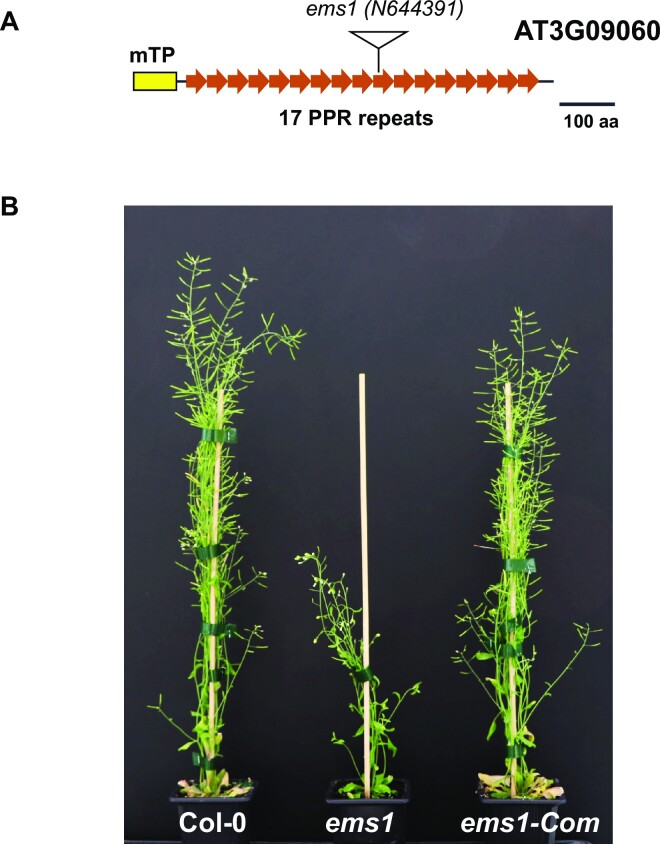
Arabidopsis *ems1* mutant displays a retarded growth phenotype. (**A**) Schematic representation of the EMS1 protein with 17 putative PPR repeats and the site of T-DNA insertion in the *ems1* mutant (N644391). The mitochondrial transit peptide is denoted as mTP. (**B**) Photograph of 5-week-old Arabidopsis plants showing the stunted growth phenotype of *ems1* mutants compared to the wild type (Col-0) and a fully-complemented *ems1*-Com plants.

We originally identified the *ems1* mutant from a collection of Arabidopsis mutants bearing T-DNA insertions in nuclear genes encoding mitochondrial-targeted PPR proteins. The affected PPR gene corresponds to AT3G09060 which encodes a PPR protein comprising 17 canonical P-type repeats, according to the PlantPPR database (http://ppr.plantenergy.uwa.edu.au/). The T-DNA in the *ems1* mutant (SALK_144391) is located in the gene segment encoding the tenth PPR repeat, precisely at position + 1926 relative to the start codon (ATG, A = +1) (Figure 1A). No *EMS1* cDNA covering the T-DNA insertion site could be amplified from homozygous mutant plants, strongly supporting the nullity of the *ems1* mutant (Supplementary Figure S1). Compared to the wild-type, *ems1* mutant plants exhibited a slow growth phenotype throughout their development and reached about half the size of the Col-0 reference plants when grown under long-day conditions (16 h light/8 h dark) in the greenhouse (Figure 1B). A genetic complementation test was then developed to confirm that the observed mutant phenotype was due to the T-DNA insertion in the *EMS1* gene. To this end, the full-length coding sequence of *EMS1* was fused to the green fluorescent protein (GFP) tag under the control of the 35S promoter, yielding a 35S::EMS1::GFP construct that was then transformed into heterozygous *ems1* plants. The resulting homozygous transgenic *ems1* plants showed a restored wild-type phenotype (Figure 1B), supporting that the inactivation of the AT3G09060 gene is responsible for the defective phenotype of the *ems1* mutant.

### The EMS1 protein is targeted to mitochondria *in vivo*

**Figure 2.**
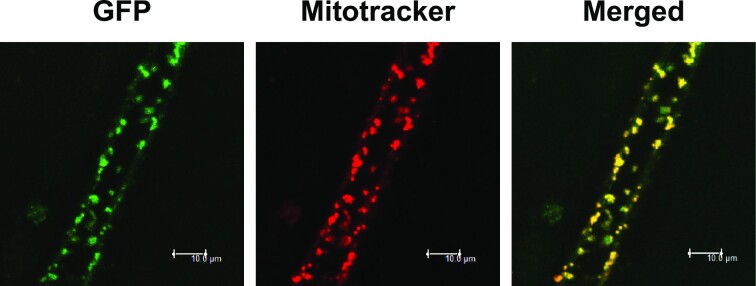
The EMS1 gene encodes a mitochondrially targeted PPR protein. Confocal microscopy images showing the subcellular distribution of an EMS1::GFP translational fusion in the root cells of complemented *ems1* transgenic plants (Figure 1B). The left panel shows the green fluorescence of GFP. The center panel displays the Mitotracker Red revealed red fluorescence of mitochondria. The merged signal is displayed on the right. Scale bars: 10 μm.

Based on the Arabidopsis subcellular database SUBA (17), the EMS1 protein harbors a putative mitochondrial targeting sequence. To verify its subcellular distribution, the *in vivo* localization of the EMS1::GFP translational fusion expressed in the *ems1* complemented plants (Figure 1B) was analyzed by confocal microscopy. The resulting GFP fluorescence signal was found to co-localize with the red signals of the MitoTracker (Figure 2), thus confirming the mitochondrial localization of the EMS1 protein *in vivo*.

### Impaired complex I accumulation in *ems1* mutants

**Figure 3.**
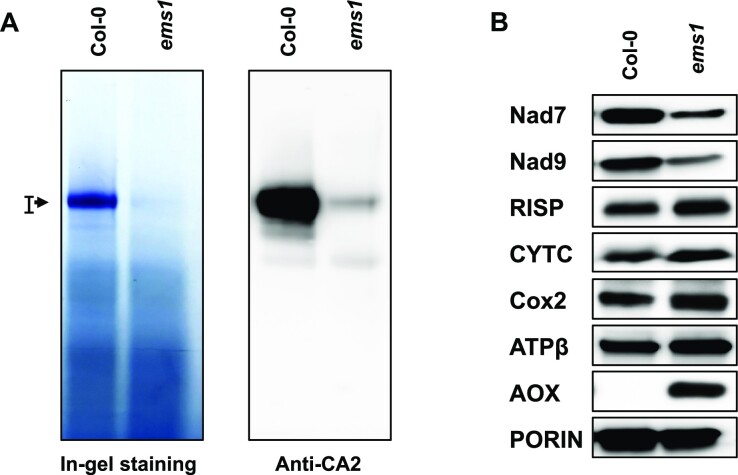
Respiratory complex I biogenesis is impaired in the *ems1* mutant. (**A**) Mitochondrial complex I accumulation was analyzed in *ems1* and Col-0 plants using BN-PAGE gel. In-gel staining revealing the NADH dehydrogenase activity of complex I is shown in the left panel. The right panel shows the detection of complex I by immunoblot assay with antibodies against the mitochondrial CA2 (carbonic anhydrase2). The holocomplex I is indicated by an arrowhead. Equal loading of the two samples was confirmed by Coomassie blue staining of an equivalent BN-PAGE gel shown in Supplementary Figure S3. (**B**) Steady-state levels of mitochondrial proteins were determined by SDS-PAGE. Total mitochondrial protein extracts were prepared from the indicated genotypes and probed with antibodies against subunits of respiratory complex I (Nad7 and Nad9), complex III (RISP), complex IV (Cox2), complex V (ATPβ), cytochrome *c* (CYT *c*) and alternative oxidase (AOX). Porin was used as a protein loading control.

The mitochondrial localization of the EMS1 protein encouraged us to consider that the developmental alterations of *ems1* mutants might result from impaired respiratory activity in these plants. The accumulation and activity of the different respiratory complexes were thus examined by blue native polyacrylamide gel electrophoresis (BN-PAGE). We observed a dramatic reduction in complex I accumulation in the *ems1* mutant, both by in-gel activity staining and immunoblot analysis using an antibody against the carbonic anhydrase 2 (CA2), a complex I subunit (Figure 3A). The observed reduction in complex I was restored in complemented plants, confirming that it was associated with inactivation of the *EMS1* gene (Supplementary Figure S2). We did not detect any reduction in the accumulation of other respiratory complexes (complexes III, IV and V), which in fact slightly overaccumulate in the mutant as compared to the wild type (Supplementary Figure S3).

The steady-state levels of several subunits of respiratory complexes were also analyzed by immunoblot assay. As shown in Figure 3B, two subunits of complex I (NADH DEHYDROGENASE 7 and 9 (Nad7 and Nad9)) were reduced in the *ems1* mutant, which is consistent with the decreased abundance and activity of complex I in these plants (Figure 3A). Other proteins such as RISP (a subunit of complex III), CYTC (a mobile protein that shuttles between complex III and IV), Cox2 (a subunit of complex IV), and ATP-β (a subunit of complex V) accumulated at slightly higher or near normal levels in mutant plants compared to the wild type. The results are consistent with the abundance of corresponding respiratory complexes, as revealed by BN-PAGE gel analysis (Supplementary Figure S3). The alternative respiratory pathway is known to be induced when the mitochondrial electron transport chain is compromised. As expected, the steady-state level of alternative oxidase (AOX) protein was found to be strongly increased in the *ems1* mutant (Figure 3B). Taken together, our results show that the loss of EMS1 activity affects the biogenesis of mitochondrial complex I, which in turn leads to the activation of the alternative respiratory pathway.

### EMS1 plays a role in the stability of the *nad2* exon 1-2 precursor transcript

**Figure 4.**
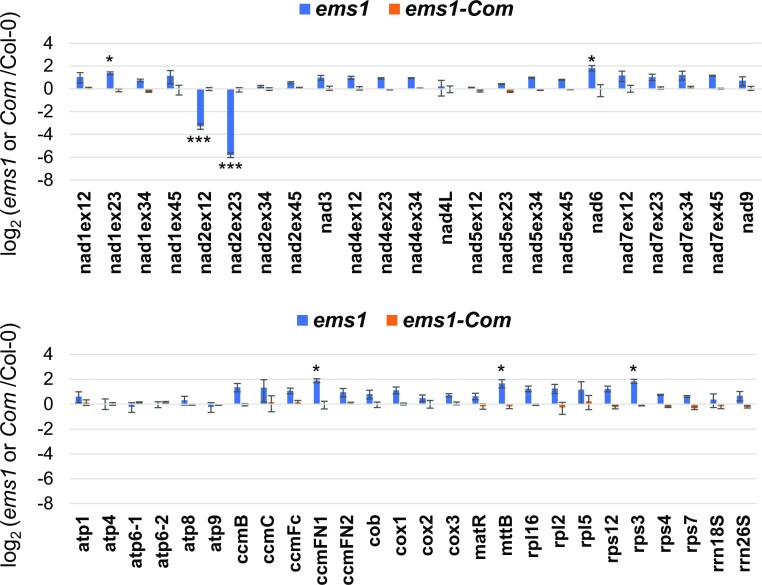
*nad2* transcripts spliced for introns 1 and 2 accumulate at reduced levels in the *ems1* mutant. The steady-state levels of mature mitochondrial mRNAs were analyzed by RT-qPCR in wild-type (Col-0) and *ems1* plants. The log_2_ ratios of transcript accumulation in *ems1* to wild-type are presented. For mRNAs without introns, a single RT-qPCR was performed, while for intron-containing transcripts, the accumulation of spliced transcript for each intron was examined. The analysis was based on three biological and three technical replicates per genotype, and standard errors are indicated. The data were normalized to the nuclear 18S rRNA gene. Significant differences are indicated with one (*P* < 0.05) or three (*P* < 0.001) asterisks.

**Figure 5.**
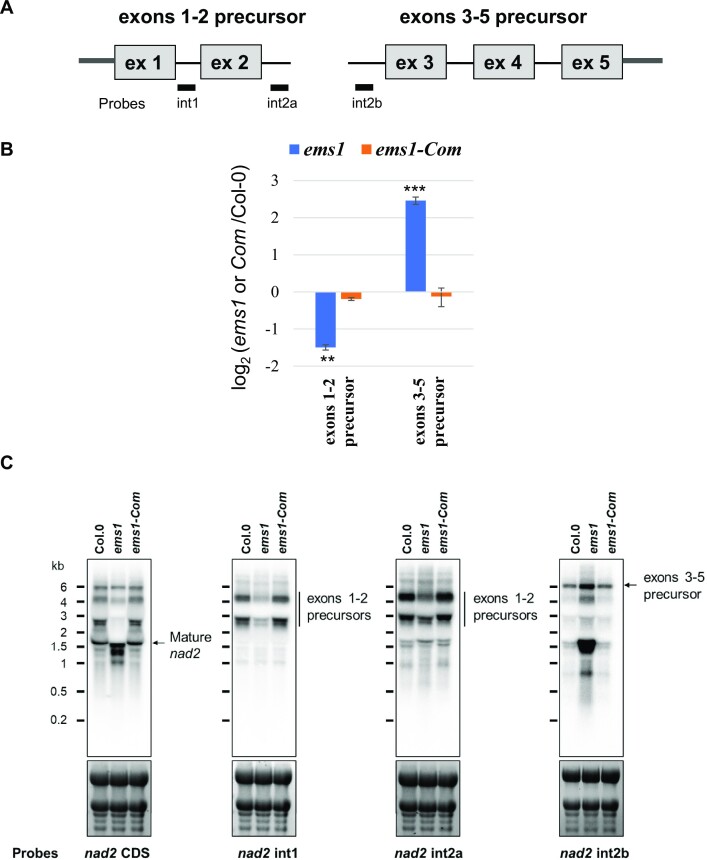
*nad2* exons 1–2 precursor transcripts are destabilized in the *ems1* mutant. (**A**) Diagram of *nad2* exons 1–2 and *nad2* exons 3–5 precursor transcripts that are joined by a *trans*-splicing reaction involving *nad2* intron 2 to form the mature *nad2* mRNA. Boxes represent exons. Introns and 5′ and 3′ UTRs are shown as thick and thin lines, respectively. Probes used for RNA gel blots are also indicated (int1, int2a, int2b). **(B**) RT-qPCR analysis measuring the steady-state levels of *nad2* exons 1–2 and *nad2* exon 3–5 precursor RNAs in complemented plants (*ems1*-Com) and *ems1* plants. The histograms show log_2_ ratios of transcript accumulation levels in *ems1* and complemented plants (*ems1*-Com) plants to wild-type. Three biological replicates and three technical replicates were used per genotype. Standard errors are indicated. Significant differences are indicated with two (*P* < 0.005) or three (*P* < 0.001) asterisks. (**C**) RNA gel blots showing the accumulation profiles of *nad2* transcripts in the *ems1* mutant compared to Col-0 and *ems1*-Com plants. Probes used for hybridization are indicated below blots. Ethidium bromide staining of ribosomal RNAs is shown below the blots and serves as a loading control. The band of similar size to that of mature *nad2* detected in *ems1* plants corresponds to the exon 3–5 precursor spliced for introns 3 and 4. The two bands corresponding to the exons 1–2 precursors differ by the presence or absence of the co-transcribed *rps4* gene (31).

P-type PPR proteins are known to be involved in a wide range of RNA processing events in plant organelles (3,18). The complex I deficiency in *ems1* mutant lets us to hypothesize that EMS1 protein could have a role in the expression of one or several mitochondria-encoded mRNAs encoding a complex I subunit. To this end, we first compared the steady-state levels of mature mitochondrial transcripts by RT-qPCR in *ems1* mutant and Col-0 reference plants. Most mature mitochondrial mRNAs were found to accumulate at the same or slightly higher levels in *ems1* compared to the wild type except for the RNA species spliced for *nad2* intron 1 and intron 2 whose steady-state levels were greatly reduced in *ems1* plants (Figure 4, RT-qPCR *nad2* ex1-2 and *nad2* ex2-3). The maturation of the Arabidopsis *nad2* mRNA results from the fusion of two distinct pre-mRNA molecules (called *nad2* exons 1–2 and *nad2* exon 3–5, see Figure 5) that are reunified by a *trans*-splicing reaction implicating the two halves of *nad2* intron 2, named 2a and 2b (Figure 5A). To understand what causes the reduction in *nad2* intron 1 and intron 2 splicing in *ems1* plants, the steady level of these two precursor mRNAs was evaluated by RT-qPCR. This approach revealed opposite changes in the abundance of these two precursors, with an overaccumulation of *nad2* exons 3–5 pre-mRNAs and an underaccumulation of na*d*2 exons 1–2 precursors compared to the wild type (Figure 5B). RNA gel blot analysis confirmed these observations (Figure 5C). Such opposite behavior of the two precursor RNAs bracketing *nad2* intron 2 did not support a role for EMS1 in the splicing of this intron. Effectively, altered intron splicing is suspected when all involved precursor transcripts overaccumulate (since they are less utilized in the splicing reaction) and the corresponding mature mRNA decreases in abundance accordingly. Instead, it suggested that the loss of mature *nad2* mRNA was most likely due to instability of *nad2* exons 1–2 precursors rather than a splicing defect of *nad2* intron 2 itself. The overabundance of *nad2* exons 3–5 precursor RNAs is consistent with this hypothesis since they cannot be processed in the *trans*-splicing reaction involving *nad2* intron 2 in the absence of the *nad2* exons 1–2 precursors. Overall, these observations strongly suggested that the EMS1 protein plays most likely a key role in the stabilization of *nad2* exons 1–2 precursor RNAs and not in the *nad2* intron 2 splicing reaction *per se*.

### The EMS1 protein binds within the 3′ region of the *nad2* exons 1–2 precursor transcript

**Figure 6.**
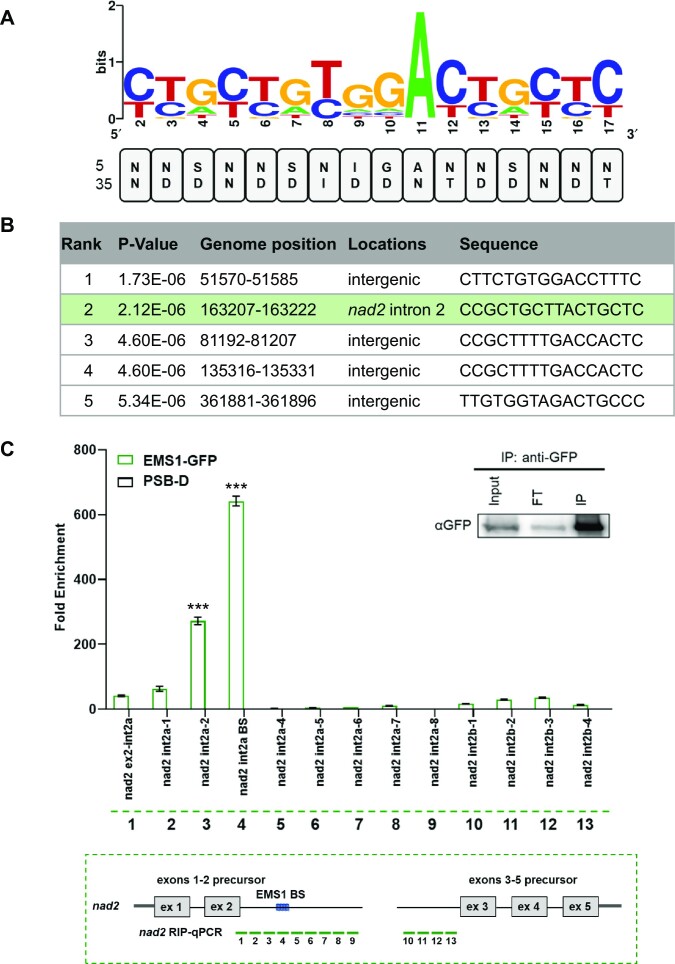
The EMS1 protein specifically associates with 3′ end region of the *nad2* exons 1–2 precursor *in vivo*. (**A**) Prediction of the EMS1 binding site according to the PPR code. The amino acid at positions 5 and 35 of each EMS1 PPR repeat were extracted and are listed from N to C-terminus. The probabilities of nucleotide recognition by each PPR repeat were calculated according to the PPR code and depicted as a sequence logo obtained with http://weblogo.berkeley.edu/. (**B**) The obtained recognition motif was then used to scan both strands of the non-edited and edited Arabidopsis mitochondrial genome from Col-0. The five most-probable EMS1 binding sequences are shown with their *P*-values determined by FIMO program. The green box marks the site located in the 3′ region of 5′-half of *nad2* intron 2, which was shown to be the RNA target of EMS1 in subsequent experiments. (**C**) Analysis of cDNAs corresponding to RNAs coimmunoprecipitated with EMS1-GFP in transgenic Arabidopsis cell lines. Total extracts were immunoprecipitated with anti-GFP antibody and untransformed PSB-D cells were used as a negative control. Coimmunoprecipitated RNAs were analyzed by RT-qPCR using primers for the indicated mitochondrial transcript regions. The green dotted box below the histogram shows the different amplification products covering *nad2* intron 2a and 2b. Highest enrichment was obtained with the primer pair covering the putative EMS1 binding site (nad2 int2a BS; PCR #4). Immunoblot results of total extracts (Input), flow-through (FT), and immunoprecipitated (IP) fractions using the GFP antibody are shown at the top right of the figure panel. Significant differences are indicated with three (*P* < 0.001) asterisks.

To investigate a potential role of EMS1 in the stabilization of the *nad2* exons 1–2 precursor, its RNA binding site was predicted using the previously established PPR recognition code (19,20). We applied the PPR recognition code to EMS1 repeats 2–17 and obtained a degenerate RNA recognition sequence covering all potential EMS1 binding sites (Figure 6A). The obtained sequence was then used to scan the unedited and fully edited Arabidopsis mitochondrial genome. Interestingly, among the five most likely identified binding sites, one (CCGCUGCUUACUGCUC) involved the *nad2* transcript. This site was located in the first half of *nad2* intron 2, while the other sites were mapped to non-coding regions (Figure 6B). To determine whether EMS1 associates with this potential RNA target *in vivo*, RNA immunoprecipitation (RIP) followed by RT-qPCR assays were performed on extracts prepared from the Arabidopsis cell line expressing the 35S::EMS1::GFP fusion, which proved to be functional (Figure 1B). The EMS1-GFP fusion protein was immunoprecipitated with a GFP antibody and co-enriched RNAs were purified from the coimmunoprecipitate. The resulting RNAs were then used for cDNA synthesis and analyzed by qPCR using a set of primer pairs spanning *nad2* intron 2a and 2b (Figure 6C). Of all the regions tested, two showed highly significant enrichment, of which the one containing the predicted EMS1 binding sites was the most enriched (Figure 6C). In addition, no co-IP enrichment was observed for any other mitochondrial introns, nor for any of the ribosomal RNAs or the *nad9* mRNA whose encoded protein steady-state level was found to be reduced in *ems1* plants (Supplementary Figure S4). These data provided strong support for the binding of EMS1 to the CCGCUGCUUACUGCUC sequence that is found 815 bases downstream of *nad2* exon 2.

### The 3′ ends of *nad2* exons 1–2 precursors coincide with the EMS1 binding site

**Figure 7.**
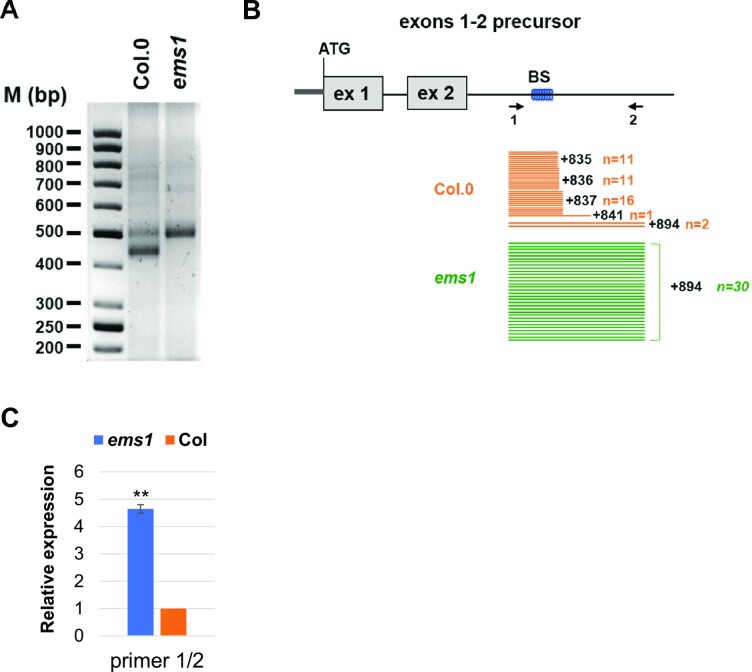
Longer *nad2* exons 1–2 precursors accumulate in the *ems1* mutant compared to the wild type. (**A**) Agarose gel showing circular-RT PCR amplification products obtained for *nad2* exons 1–2 precursor transcripts in wild-type (Col-0) and *ems1* mutants. M: DNA size ladder. (**B**) Mapping of the 3′ extremities of *nad2* exons 1–2 precursors detected in the wild-type (Col-0) and the *ems1* mutant. Orange and green bars represent sequencing results from individual circular-RT PCR clones derived from Col-0 and the *ems1* mutant, respectively. Each bar represents a single sequenced clone. The positions of the primers used for the quantitative RT-PCR analysis shown in panel C are indicated. (**C**) Abundance of long (+894) transcripts as measured by qRT-PCR in Col-0 and *ems1* mutant using the 1/2 primer set. Significant differences are indicated with two (*P* < 0.005) asterisks.

The cause of the destabilization of the *nad2* exons 1–2 precursor RNA in *ems1* plants was further investigated by comparative mapping of its 3′ extremity by circular RT-PCR in both *ems1* and wild-type plants. Agarose gel analysis of the obtained amplification products revealed two bands of different size and intensity in Col-0 plants, whereas only the larger one was detected in the *ems1* mutant (Figure 7A). Cloning and sequencing of the bands obtained in Col-0 plants showed that the small band corresponded to *nad2* exons 1–2 transcripts terminating 835–837 nucleotides downstream of exon 2 (Figure 7B) and that the larger band corresponded to longer *nad2* exons 1–2 transcripts ending 894 nucleotides downstream of exon 2. Sequence analysis of the band obtained in *ems1* showed that it corresponded to the longer form (+894) of the *nad2* exons 1–2 transcripts (Figure 7B). Quantification of the long transcripts by RT-qPCR showed that they are almost five times more abundant in the *ems1* mutant compared to wild-type (Figure 7C). Taken together, our results revealed that the *nad2* exons 1–2 precursors carry 3′ ends terminating either at position +835/+837 or +894 downstream of exon 2. The production of the short transcripts is EMS1 dependent and their 3′ ends coincide with the EMS1 RNA binding site.

## DISCUSSION

### The EMS1 protein is involved in the 3′-end formation of the 5′-half of *nad2* intron 2

The recombinogenic nature of plant mitochondrial genomes makes them prone to frequent sequence rearrangements, sometimes resulting in gene fragmentation when recombination occur within gene sequences (21). The most common type of gene splitting event results from breaks within intron sequences, resulting in the production of independently transcribed gene fragments. To reassemble a single, functional mature mRNA, the separate transcripts are joined back together by base-pairing interactions between the two intron halves, reforming a functional intron (22,23). Such *trans*-splicing reaction can only occur if the 5′ and 3′ half introns can re-associate into a splicing-competent structure. Therefore, the 5′ and 3′ intron halves must be processed and trimmed to eliminate counterproductive 3′ and 5′ RNA extensions, respectively. Our analysis of EMS1 strongly suggests that this protein plays a role in such processes by both processing and stabilizing the 3′ end of pre-mRNAs carrying the 5′ half of *nad2* intron 2. We effectively observed that *ems1* plants are impaired in the production of *nad2* transcripts spliced for intron 1 and 2, which are *cis*- and *trans*-introns, respectively. However, the lack of over-accumulation of the unspliced precursor carrying the 5′ half of *nad2* intron 2 was not in favor of a role of EMS1 in the splicing of this transcript but rather in its stabilization. A similar conclusion was drawn from the analysis of the Arabidopsis 3′-end processing PPR protein MTSF3, in which the destabilization of the *nad2* exons 3–5 precursor in the corresponding mutant results, albeit indirectly, in the abolition of intron 2 splicing (5). This hypothesis was next supported by the binding site of EMS1 which coincides with the 3′ extremity of the *nad2* exons 1–2 transcript. By sitting on mRNA termini, PPR proteins were found to be able to act as physical barriers and thereby impede the progression of exonucleases along transcripts (9). In fact, plant mitochondrial transcription termination is known to show little or no modulation and can extend for several kilobases downstream of genes (1). Thus, unnecessary 3′ sequence extensions require exonucleolytic trimming *via* the action of 3′-to-5′ exoribonucleases such as the PNPase or the RNase R homolog 1 to produce mature RNAs (24–26). In plant mitochondria, the type of transcripts stabilized by association with protective PPR proteins at their 3′ end includes mRNAs (5,10,12), but we have recently shown that precursor transcripts undergoing a *trans*-splicing reaction are similarly processed (11). EMS1 is indeed the second reported example of a pre-mRNA-protecting PPR protein, but its action in 3′ end formation does not appear to only involve the blocking of 3′ to 5′ exonucleolytic trimming. In other previously reported cases the loss of the protecting PPR protein necessarily destabilizes the target RNA and, most logically, 3′-trimmed degradation RNA products are detected in the corresponding mutants (10). In the *ems1* mutant, no 3′ shorter but 3′ longer *nad2* exons 1–2 precursors were detected (Figure 7A and B). We found that these longer transcripts accumulate at much lower levels in wild-type than in *ems1* mutant plants, suggesting that they have a certain degree of stability (Figure 7C). The increased accumulation of elongated *nad2* exons 1–2 pre-mRNAs in *ems1* plants compared to the wild type suggests that EMS1 plays a role in their shortening, most likely by inducing endonucleolytic cleavage between positions + 835 and + 894, which must be followed by exonucleolytic trimming up to the EMS1 binding site. We also observed that longer transcripts (+894) accumulate to much lower levels than + 835 precursors. The long transcripts thus appear to be inefficiently stabilized; enough nonetheless to be detected, but not to produce physiological levels of *nad2* exons 1–2 precursors and thus Nad2 protein. Therefore, the 3′-end formation of *nad2* exons 1–2 transcripts appears to involve both endonucleolytic and exonucleolytic processing events, a mechanism of 3′-end formation not previously reported in plant mitochondria.

### Analysis of the 3′ end processing of transcripts carrying other 5′-half introns in arabidopsis

**Figure 8.**
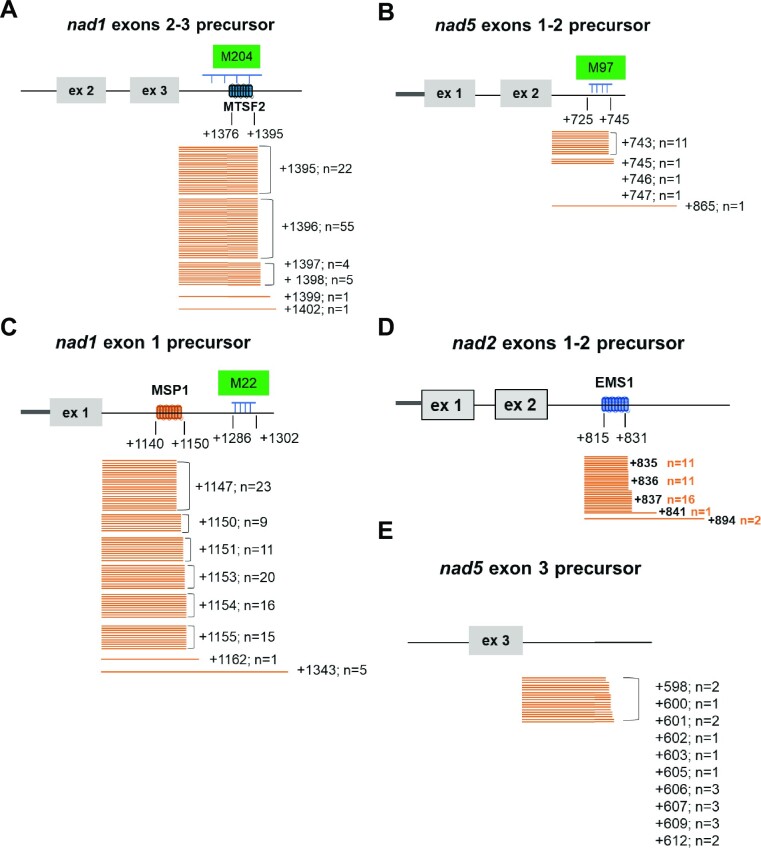
Mapping of the 3′-ends of the five 5′-half-intron-containing transcripts in Arabidopsis. The positions of the 3′ ends were determined by cloning of circular RT-PCR amplification products and sequencing of individual clones. 3′ ends are positioned relatively to the last base of the upstream exon, and the number of clones (n) corresponding to each identified end is given. The position of known stabilizing PPR proteins (EMS1, MTSF2 and MSP1) and nearby cos-RNAs (M204, M97 and M22) are indicated.

Five *trans*-spliced introns including *nad1* introns 1 and 3, *nad2* intron 2, and *nad5* introns 2 and 3 are found in Arabidopsis (23). To see if the definition of the 3′ end of 5′-half introns follows the same molecular rules, the 3′ termini of the corresponding precursor transcripts were mapped by circular RT-PCR. We also searched for the presence of short RNA footprints near the identified 3′ termini in the clustered organellar sRNA (cosRNA) database (27), which may indicate stabilization of the corresponding transcripts by binding of PPR-like proteins to their ends. Effectively, the binding of RNA stability factors, such as PPRs, has been shown to result in the accumulation of short RNA sequences corresponding to their binding sites in plant organelles (5,10,11,27–29). As shown previously, a cosRNA footprint can be found at the end of the *nad1* exons 2–3 precursor, which perfectly coincides with both the 3′ end of the transcript and the binding site of the MTSF2 PPR, ensuring the stability of the molecule (Figure 8A, (11)). Our analysis supports a similar type of stability and processing mechanism for the precursor carrying the 5′ half of *nad5* intron 2, for which the identified 3′ end overlaps with a cosRNA footprint (M97) (Figure 8B). For the three other *trans*-intron precursors, the situation appears to be less straightforward, potentially implying different 3′ end processing mechanisms. It first concerns the *nad2* exons 1–2 precursor, for which no cosRNA matches its 3′ end and the binding site of EMS1. The lack of cosRNA in this region may reflect a weaker binding of EMS1 to the transcript it protects compared to other stabilizing PPRs, such as MTSF2. A similar situation is found for the *nad1* exon 1 precursor, stabilized by the MSP1 PPR protein (30). Although a cosRNA (M22) is found in the 5′ half of *nad1* intron 1, it is located 136 bases downstream of the 3′ end of the precursor and thus the MSP1 binding site (Figure 8C). We also found some variability regarding the 3′-end of the *nad1* exon 1 precursor, suggesting imperfect transcript end protection by MSP1. The presence of a cosRNA (M22) downstream of MSP1 suggests, as for EMS1, that a two-step mechanism involving MSP1-mediated endoribonucleolytic cleavage followed by possible 3′ to 5′ trimming up to the MSP1 binding site may be responsible for the formation of the 3′ end of the *nad1* exon 1 pre-mRNA. The lack of cosRNA coinciding with both EMS1 and MSP1 binding sites may be indicative that they act similarly in 3′ end RNA processing. Our analysis also reveals an unclear 3′-end processing mechanism for the transcript carrying the 5′-half of *nad5* intron 3. Effectively, variable 3′ termini spanning over 14 bases were mapped at the end of *nad5* exon 3 precursors and no cosRNA matching with these extremities could be identified (Figure 8D), potentially excluding a protein-based stabilization mechanism for that transcript. The sequence conservation of 5′-half introns in angiosperms was next investigated in relation with the binding sites of PPR proteins involved in their stabilization. First, we found that the EMS1 protein and its RNA binding site are quite well conserved in angiosperms (Supplementary Figure S5). We also observed that the sequence of the 5′-half introns is globally conserved in angiosperms up to a region corresponding to the binding site of the factors responsible for their stability, although sequence insertions are sometimes found just downstream of the last exon in some species (Supplementary Figure S6). This break in DNA homology passing the binding site of stabilizing PPR proteins suggests that these proteins are involved in protecting the integrity of 5′-half-intron sequences, since any recombination event occurring upstream of their binding site would result in unstable transcripts. However, the situation is different for the 5′ half intron located downstream of *nad5* exon 3, as the mapped 3′ ends are not perfectly conserved in angiosperms as in other 5′ half introns, further suggesting that the stability of this transcript may not be mediated by the binding of a PPR-like protein (Supplementary Figure S6D). It thus appears that different series of events account for the 3′-end processing and the stabilization of 5′-half intron precursors in plant mitochondria. The initial view was that the processing involves a protection by an RNA binding protein sitting on transcript's 3′ end, blocking exonucleolytic degradation. The conclusions drawn from the analysis of the EMS1, which may thus also apply to the precursor transcript protected by MSP1, indicate that a two-step processing mechanism comprising an endoribonucleolytic cleavage potentially followed by exonucleolytic trimming may also account for the 3′-end formation of pre-mRNAs in plant mitochondria.

## Supplementary Material

gkad493_Supplemental_File

## Data Availability

Sequence data from this article can be found in the GenBank/EMBL data libraries under accession numbers MTSF1, AT1G06710; MTSF2, AT1G52620; MTSF3, AT2G02150; MTSF4, AT4G19440; EMS1, AT3G09060; MSP1, AT4G20090.
