# Supplementary material for: Interplay of endonucleolytic and exonucleolytic processing in the 3′-end formation of a mitochondrial nad2 RNA precursor in Arabidopsis
Source: Nucleic Acids Res. 2023 Jun 9;51(14):7619–30. doi: 10.1093/nar/gkad493 (PMC10415111; doi:10.1093/nar/gkad493)
Supplement: gkad493_Supplemental_File [file gkad493_supplemental_file.pdf]

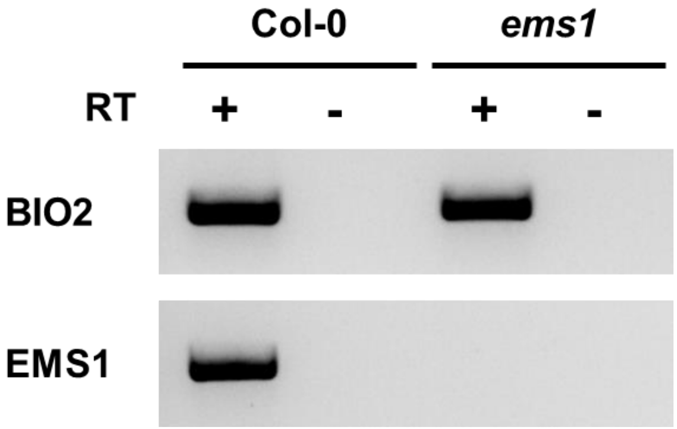

Supplementary Figure S1: RT-PCR analysis of the *EMS1* transcript in Col-0 and *ems1* plants. PCR amplification was performed on cDNA prepared from both wild-type and *ems1* plants using primers located on both sides of the T-DNA insertion site corresponding to the *ems1* mutation. The amplification of *BIO2* cDNA was performed separately to assess the efficiency of the RT reaction. The initial RT reaction was conducted with or without reverse transcriptase (RT+ or RT-, respectively). The shown amplification products were size-fractionated on an agarose gel**.**

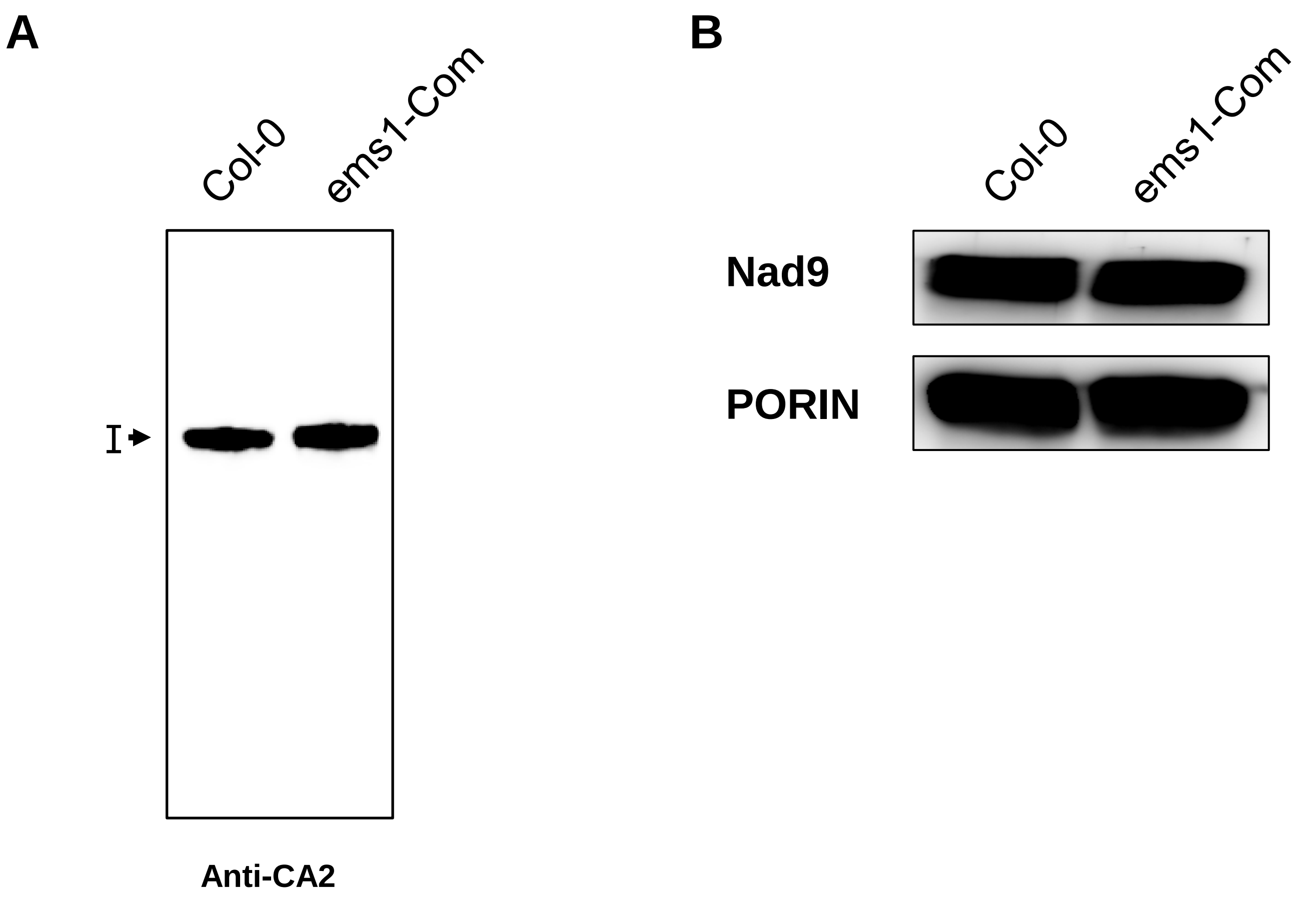

Supplementary Figure S2. Restoration of complex I accumulation to wild-type levels in *ems1* complemented plants. (A) BN-PAGE analysis of mitochondrial complex I accumulation in the *ems1* complemented plants (ems-Com) as compared to the wild type (Col-0). Complex I was detected on BN-PAGE blots using antibodies specific for the mitochondrial CA2 (carbonic anhydrase 2) subunit. Holocomplex I is indicated by an arrowhead. (B) Detection of Nad9 protein in mitochondrial extracts prepared from the *ems1* complemented and wild-type plants. Crude membrane extracts of the indicated genotypes were separated by SDS-PAGE and probed with antibodies against Nad9, a subunit of complex I. Porin was used as a protein loading control.

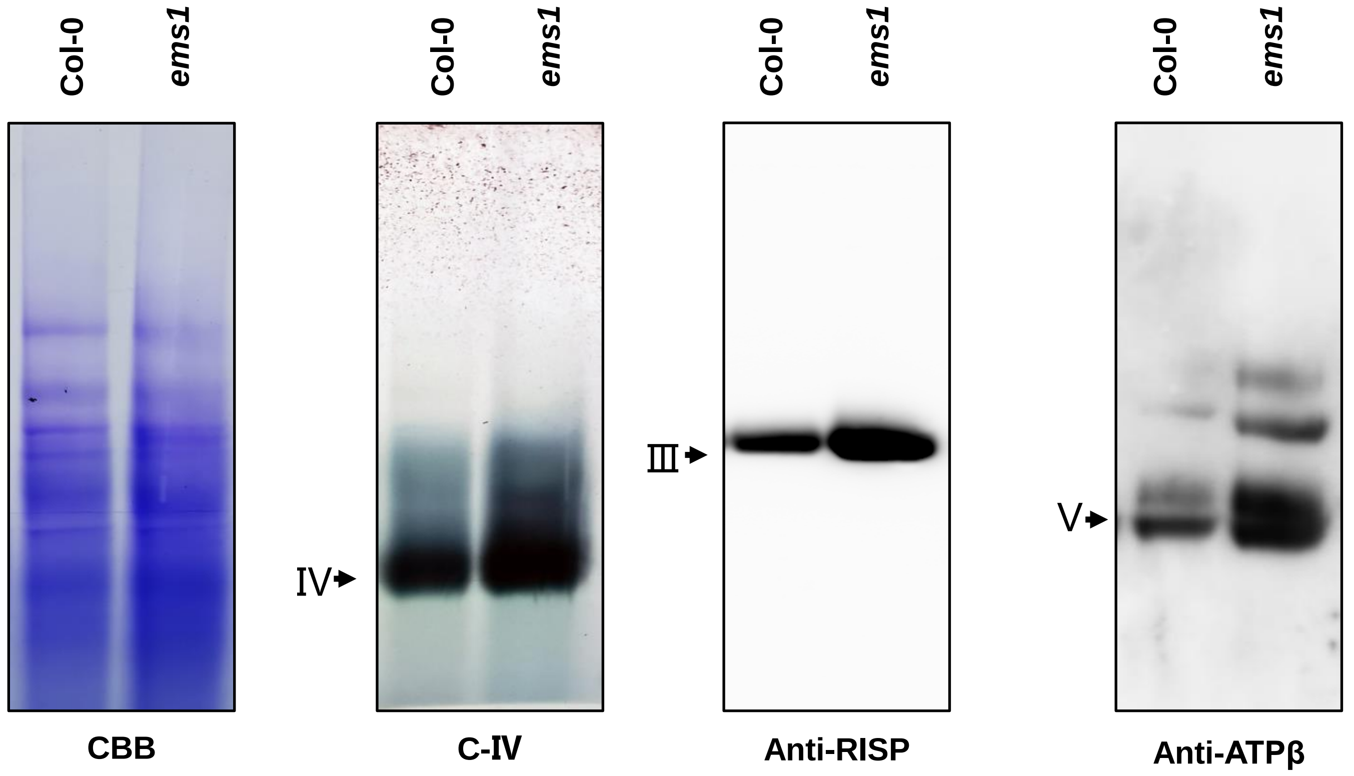

Supplementary Figure S3. BN-PAGE analysis of the respiratory chain complexes in *ems1* and Col-0 plants. After the run, BN-PAGE gels were either stained with Coomassie blue (CBB) to demonstrate equal loading of the two samples or with specific chemicals to reveal the steady-state level of complex IV (C-IV). Complex III and V were detected by hybridization of the membranes after transfer with the indicated antibodies.

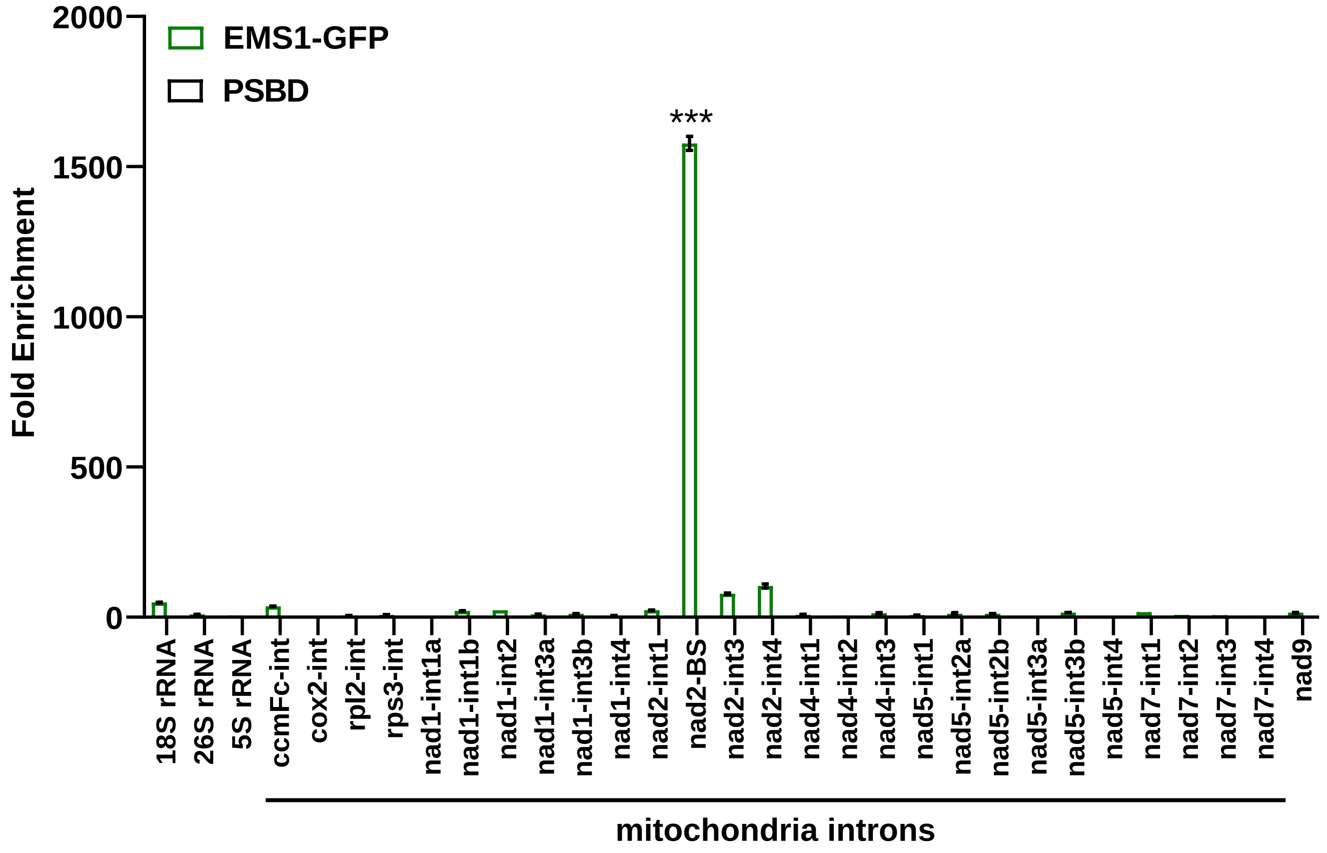

Supplementary Figure S4. RNA immunoprecipitation analysis of EMS1 target RNA *in vivo*. Analysis of cDNAs corresponding to RNAs coimmunoprecipitated with EMS1-GFP in transgenic Arabidopsis cell lines. Total extracts were immunoprecipitated with anti-GFP antibody and untransformed PSB-D cells were used as a negative control. Coimmunoprecipitated RNAs were analyzed by RT-qPCR using primers for the indicated mitochondrial transcript regions. Significant differences are indicated with three (P < 0.001) asterisks.

(A)

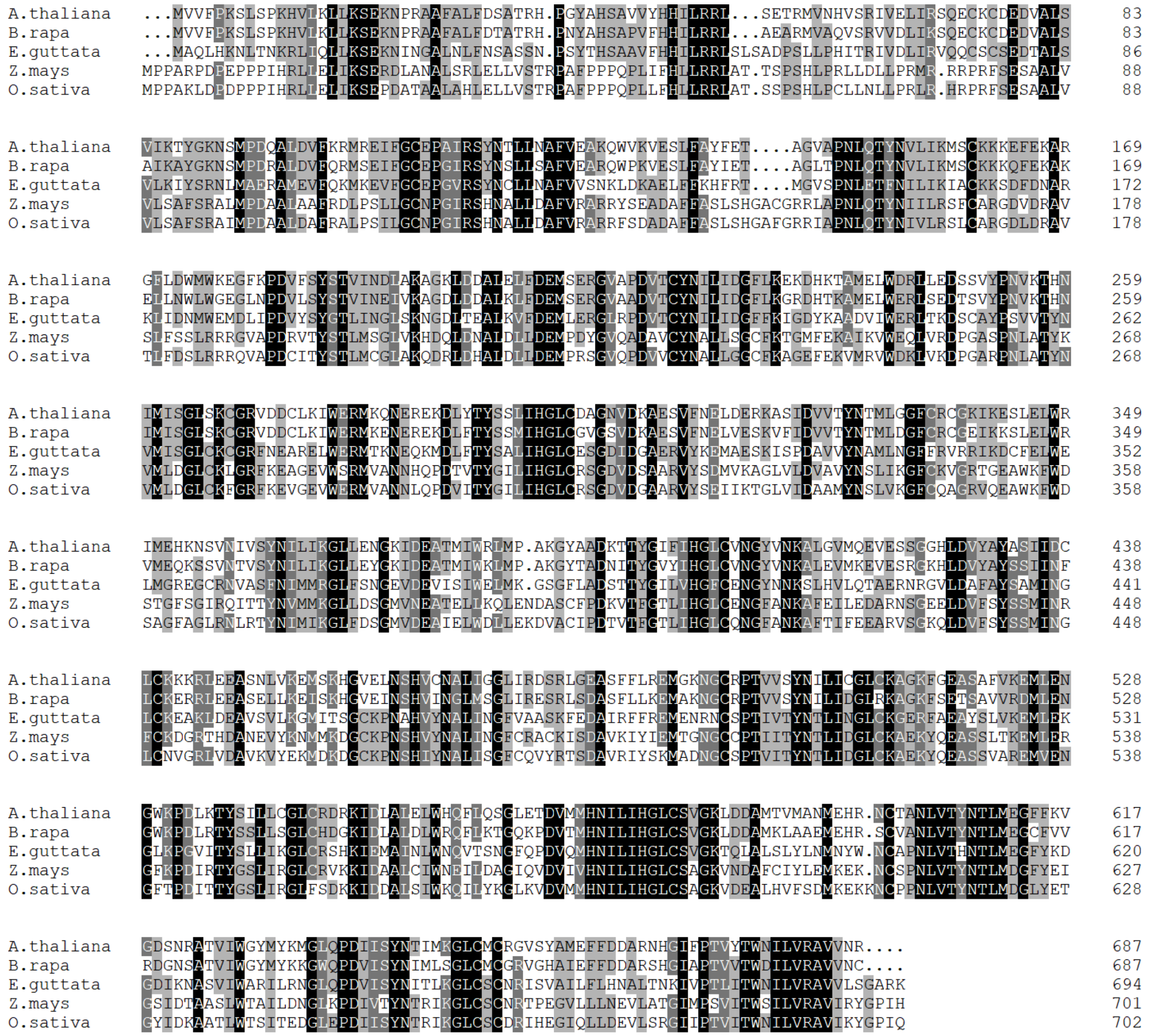
A.thaliana ...MVVFPKSLSPKHVLKLLKSEKNPRAAFALFDSATRH.PGYAHSAVVYHHILRRL...SETRMVNHVSRIVELIRSQECKCDEDVALS 83
B.rapa ...MVVFPKSLSPKHVLKLLKSEKNPRAAFALFDTATRH.PNYAHSAPVFHHILRRL...AEARMVAQVSRVVDLIKSQECKCDEDVALS 83
E.guttata ...MAQLHKNLTNKRLIQLLKSEKNINGALNLFNSASSN.PSYTHSAAVFHHILRRLSLSADPSLLPHITRIVDLIRVQQCSCSEDTALS 86
Z.mays MPPARPDPEPPPIHRLIELIKSEPRDLANALSRLELLVSTRPAFPPPQPLIPHLLRRLAT.TSPSHLPRLLDLLPRMR.RRPRFSESAALV 88
O.sativa MPPAKLDPDPPPIHRLIELIKSEPDATAALAHLELLVSTRPAFPPPQPLLFHLLRRLAT.SSPSHLPCLLNLLPRLR.HRPRFSESAALV 88
A.thaliana VIKTYGKNSMPDQALDVFKRMREIFGCEPAIRSYNTLLNAFVEAKQWVKVESLFAYFET....AGVAPNLQTYNVLIKMSCKKKEFEKAR 169
B.rapa AIKAYGKNSMPDRALDVFQRMSEIFGCEPGIRSYNSLLSAFVEARQWPKVESLFAYIET....AGLTPNLQTYNVLIKMSCKKKQFEKAK 169
E.guttata VLKIYSRNLMAERAMEVFQKMKEVFGCEPGVRSYNCLLNAFVVSNKLDKAELFFKHFRT....MGVSPNLETFNILIKIACKKSDFDNAR 172
Z.mays VLSAFSRALMPDAALAAFRDLPSLLGCNPGIRSHNALLDAFVRARRYSEADAFFASLSHGACGRRLAPNLQTYNIILRSFCARGDVDRAV 178
O.sativa VLSAFSRAIMPDAALDAFRALPSILGCNPGIRSHNALLDAFVRARRFSDADAFFASLSHGAFGRRIAPNLQTYNIVLRSLCARGDLDRAV 178
A.thaliana GFLDWMWKEGFKPDVFSYSTVINDLAKAGKLDDALELFDEMSERGVAPDVTCYNILIDGFLKEKDHKTAMELWDRLLEDSSVYPNVKTHN 259
B.rapa ELLNWLWGEGLNPDVLSYSTVINEIVKAGDLDDALKLFDEMSERGVAADVTCYNILIDGFLKGRDHTKAMELWERLSEDTSVYPNVKTHN 259
E.guttata KLIDNMWEMDLIPDVYSYGTLINGLSKNGDLTEALKVFDEMLERGLRPDVTCYNILIDGFFKIGDYKAADVIWERLTKDSCAYPSVVTYN 262
Z.mays SLFSSLRRRGVAPDRVTYSTLMSGLVKHDQLDNALDLLDEMPDYGVQADAVCYNALLSGCFKTGMFEKAIKVWEQLVRDPGASPNLATYK 268
O.sativa TLFDSLRRRQVAPDCITYSTLMCGLAKQDRLDHALDLLDEMPRSGVQPDVVCYNALLGGCFKAGEFEKVMRVWDKLVKDPGARPNLATYN 268
A.thaliana IMISGLSKCGRVDDCLKIWERMKQNEREKDLYTYSSLIHGLCDAGNVDKAESVFNELDERKASIDVVTYNTMLGGFCRCGKIKESLELWR 349
B.rapa IMISGLSKCGRVDDCLKIWERMKENEREKDLFTYSSMIHGLCGVGSVDKAESVFNELVESKVFIDVVTYNTMLDGFCRCGEIKKSLELWR 349
E.guttata VMISGLCKCGRFNEAREIWERMTKNEQKMDLFTYSALIHGLCESGDIDGAERVYKEMAESKISPDAVVYNAMLNGFFRVRRIKDCFELWE 352
Z.mays VMLDGLCKLGRFKEAGEVWSRMVANNHQPDTVTYGILIHGLCRSGDVDSAARVYSDMVKAGLVLDVAVYNSLIKGFCKVGRTGEAWKFWD 358
O.sativa VMLDGLCKFGRFKEVGEVWERMVANNLQPDVITYGILIHGLCRSGDVDGAARVYSEIIKTGLVIDAAMYNSLVKGFCQAGRVQEAWKFWD 358
A.thaliana IMEHKNSVNIVSYNILIKGLLENGKIDEATMIWRLMP.AKGYAADKTTYGIFIHGLCVNGYVNKALGVMQEVESSGGHLDVYAYASIIDC 438
B.rapa VMEQKSSVNTVSYNILIKGLLEYGKIDEATMIWKLMP.AKGYTADNITYGVYIHGLCVNGYVNKALEVMKEVESRGKHLDVYAYSSIINF 438
E.guttata LMGREGCRNVASFNIMMRGLFSNGEVDEVISIWELMK.GSGFLADSTTYGILVHGFCENGYNNKSLHVLQTAERNRGVLDAFAYSAMING 441
Z.mays STGFSGIRQITTYNVMMKGLLDSGMVNEATELLKQLENDASCFPDKVTFGTLIHGLCENGFANKAFEILEDARNSGEELDVFSYSSMINR 448
O.sativa SAGFAGLRNLRTYNIMIKGLFDSGMVDEAIELWDLLEKDVACIPDTVTFGTLIHGLCQNGFANKAFTIFEEARVSGKQLDVFSYSSMING 448
A.thaliana LCKKKRLEEASNLVKEMSKHGVELNSHVCNALIGGLIRDSRLGEASFFLREMGKNGCRPTVVSYNILICGLCKAGKFGEASAFVKEMLEN 528
B.rapa LCKERRLEEASELLKEISKHGVEINSHVINGLMSGLIRESRLSDASFLLKEMAKNGCRPTVVSYNILIDGLRKAGKFSETSAVVRDMLEN 528
E.guttata LCKEAKLDEAVSVLKGMITSGCKPNAHVYNALINGFVAASKFEDAIRFFREMENRNCSPTIVTYNTLINGLCKGERFAEAYSLVKEMLEK 531
Z.mays FCKDGRTHDANEVYKNMVKDGCKPNSHVYNALINGFCRACKISDAVKIYIEMTGNGCCPTIITYNTLIDGLCKAEKYQEASSLTKEMLER 538
O.sativa LCNVGRLVDAVKVYEKMDKDGCKPNSHIYNALISGFCQVYRTSDAVRIYSKMADNGCSPTVITYNTLIDGLCKAEKYQEASSVAREMVEN 538
A.thaliana GWKPDLKTYSILLCGLCRDRKIDLALELWHQFLQSGLETDVMMHNILIHGLCSVGKLDDAMTVMANMEHR.NCTANLVTYNTLMEGFFKV 617
B.rapa GWKPDLRTYSSLLSGLCHDGKIDLALDLWRQFLKTGQKPDVTMHNILIHGLCSVGKLDDAMKLAAEMEHR.SCVANLVTYNTLMEGCFVV 617
E.guttata GLKPGVITYSLLIKGLCRSHKIEMAINLWNQVTSNGFQPDVQMHNILIHGLCSVGKTQLALSLYLNMNYW.NCAPNLVTHNTLMEGFYKD 620
Z.mays GFKPDIRTYGSLIRGLCRVKKIDAALCIWNEILDAGIQVDVIVHNILIHGLCSAGKVNDAFCIYLEMKEK.NCSPNLVTYNTLMDGFYEI 627
O.sativa GFTPDITTYGSLIRGLFSDKKIDDALSIWKQILYKGLKVDVMMHNILIHGLCSAGKVDEALHVFSDMKEKKNCPPNLVTYNTLMDGLYET 628
A.thaliana GDSNRATVIWGYMYKMGLQPDIISYNTIMKGLCMCRGVSYAMEFFDDARNHGIFPTVYTWNILVRAVVNR.... 687
B.rapa RDGNSATVIWGYMYKKGWQPDVISYNIMLSGLCMCGRVGHAIEFFDDARSHGIAPTVVTWDILVRAVVNC.... 687
E.guttata GDIKNASVIWARILRNGLQPDVISYNITLKGLCSCNRISVAILFLHNALTNKIVPTLITWNILVRAVVLSGARK 694
Z.mays GSIDTAASLWTAILDNGLKPDIVTYNTRIKGLCSCNRTPEGVLLLNEVLATGIMPSVITWSILVRAIIRYGPIH 701
O.sativa GYIDKAATLWTSITEDGLEPDIISYNTRIKGLCSCDRIHEGIQLLDEVLSRGIIPTVITWNILVRAVIKYGPIQ 702

(B)

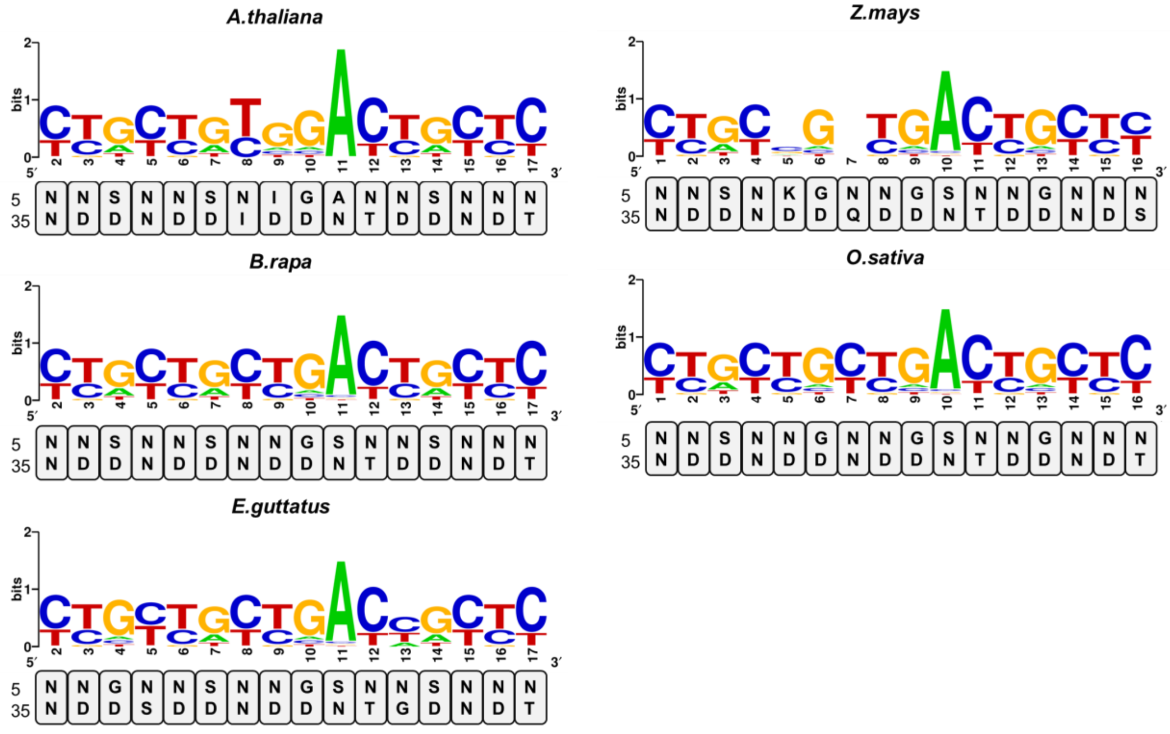
A.thaliana
Z.mays
B.rapa
O.sativa
E.guttatus
bits
5′
3′
5
35

(C)

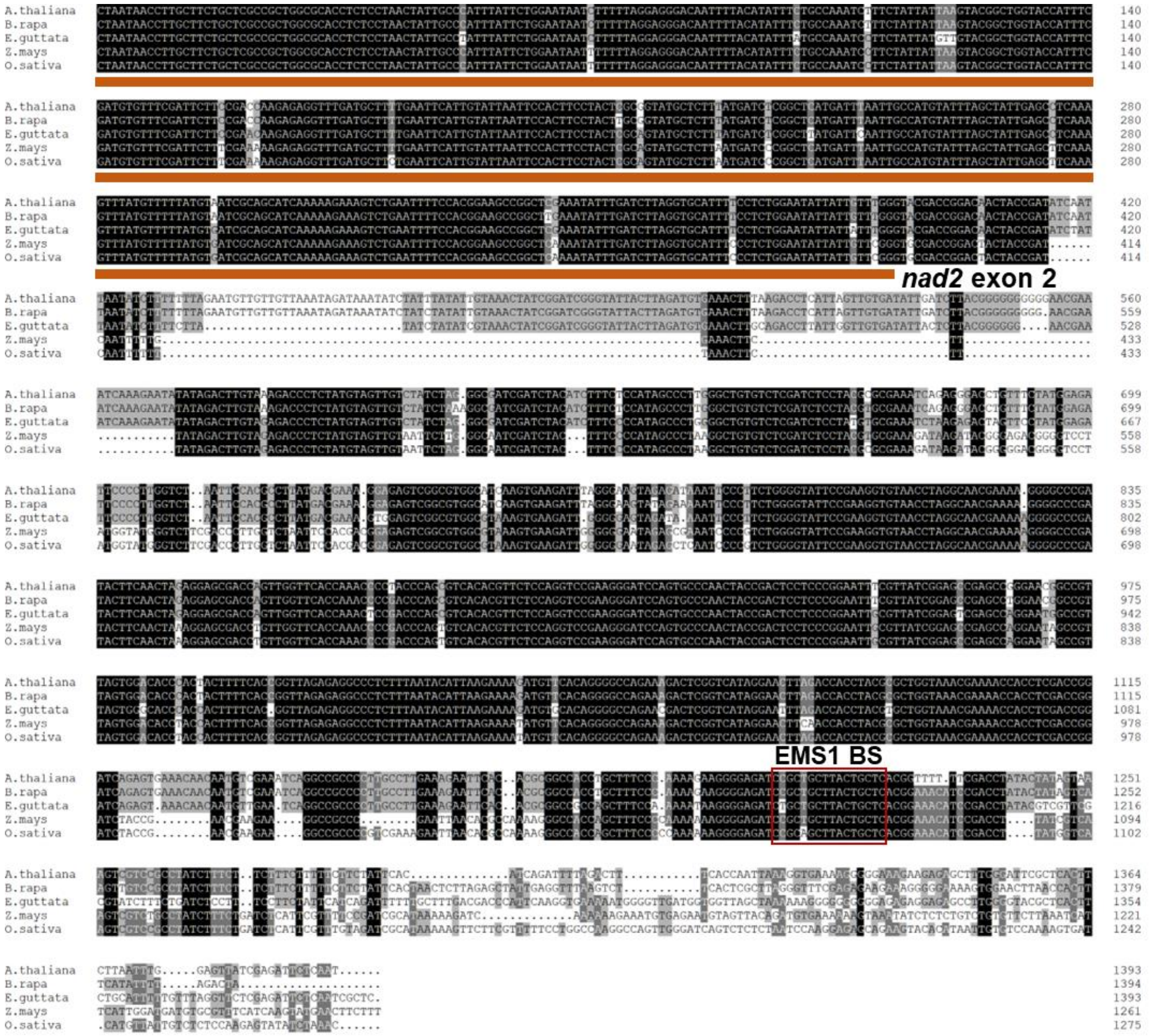

Supplementary Figure S5. The EMS1 binding site is conserved in angiosperms. (A) Multiple sequence alignment of the Arabidopsis EMS1 protein with putative orthologous proteins from a representative selection of dicots (*Brassica rapa*, *Erythranthe guttata*) and monocots (*Oryza sativa*, *Zea mays*). (B) Predicted RNA binding sites of Arabidopsis EMS1 and putative orthologous proteins from the indicated plant species. Combinations of amino acids 5 and 35 are listed from N to C-terminus. The resulting combinations were then used to calculate the probability of nucleotide recognition by each individual PPR repeat according to the PPR code. The sequence logos depicting these probabilities were obtained with http://weblogo.berkeley.edu/. (C) Multiple sequence alignment of the 3' region of the *nad2* exon 1-2 precursor from the above-mentioned plant species. The region corresponding to the EMS1 binding site (BS) is framed in red.

(A)

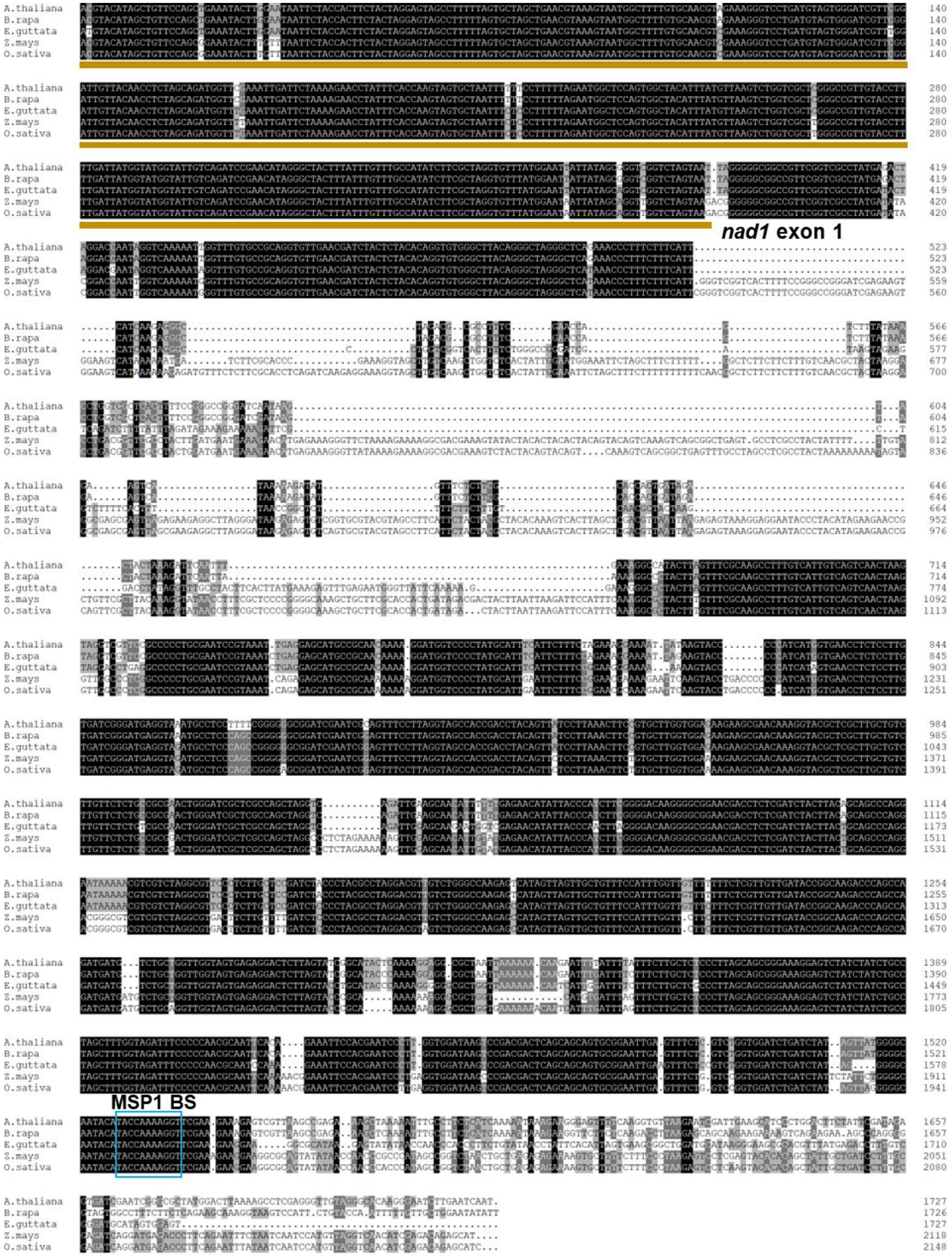

(B)

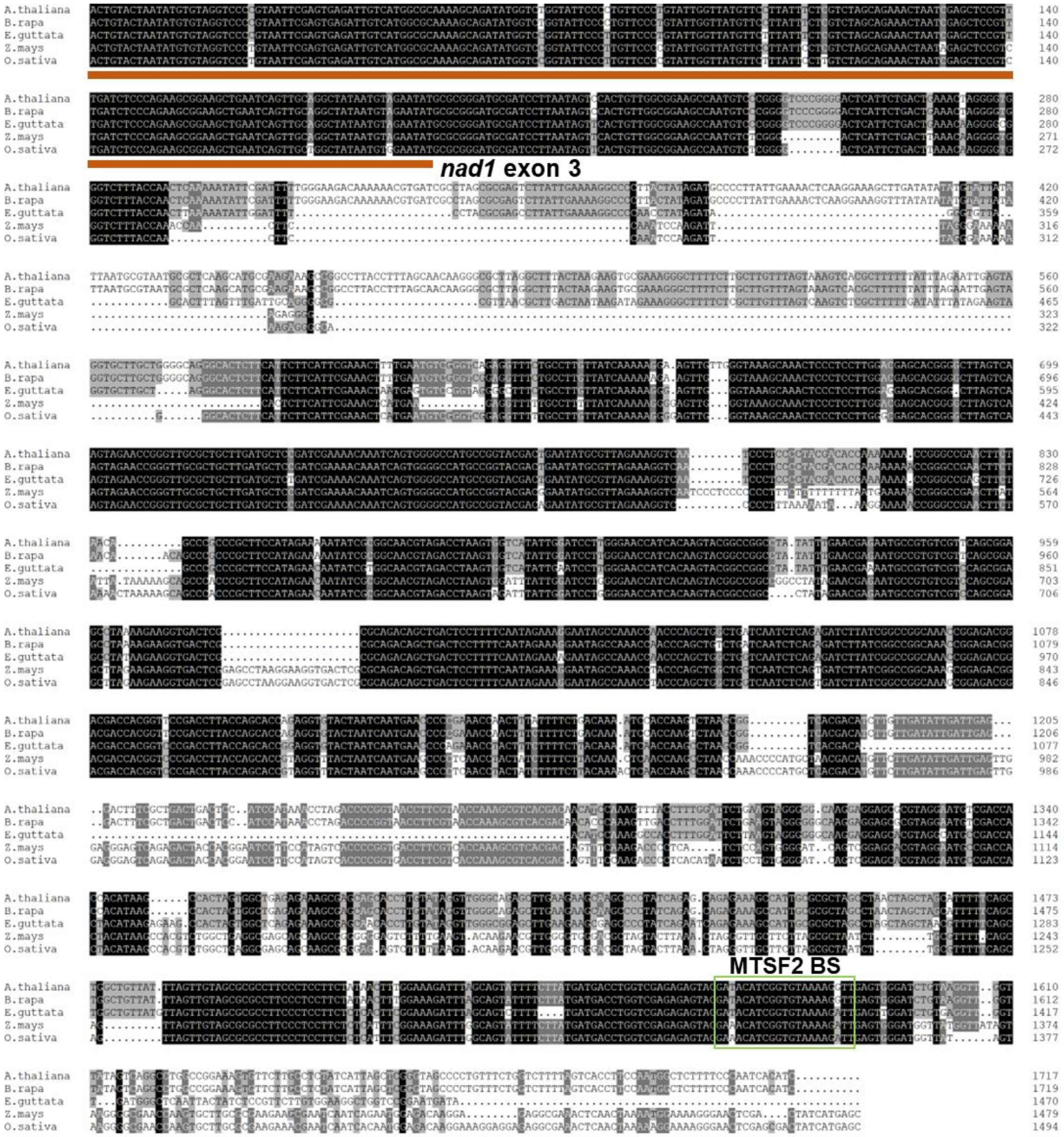
A.thaliana
B.rapa
E.guttata
Z.mays
O.sativa
nad1 exon 3
MTSF2 BS

(C)

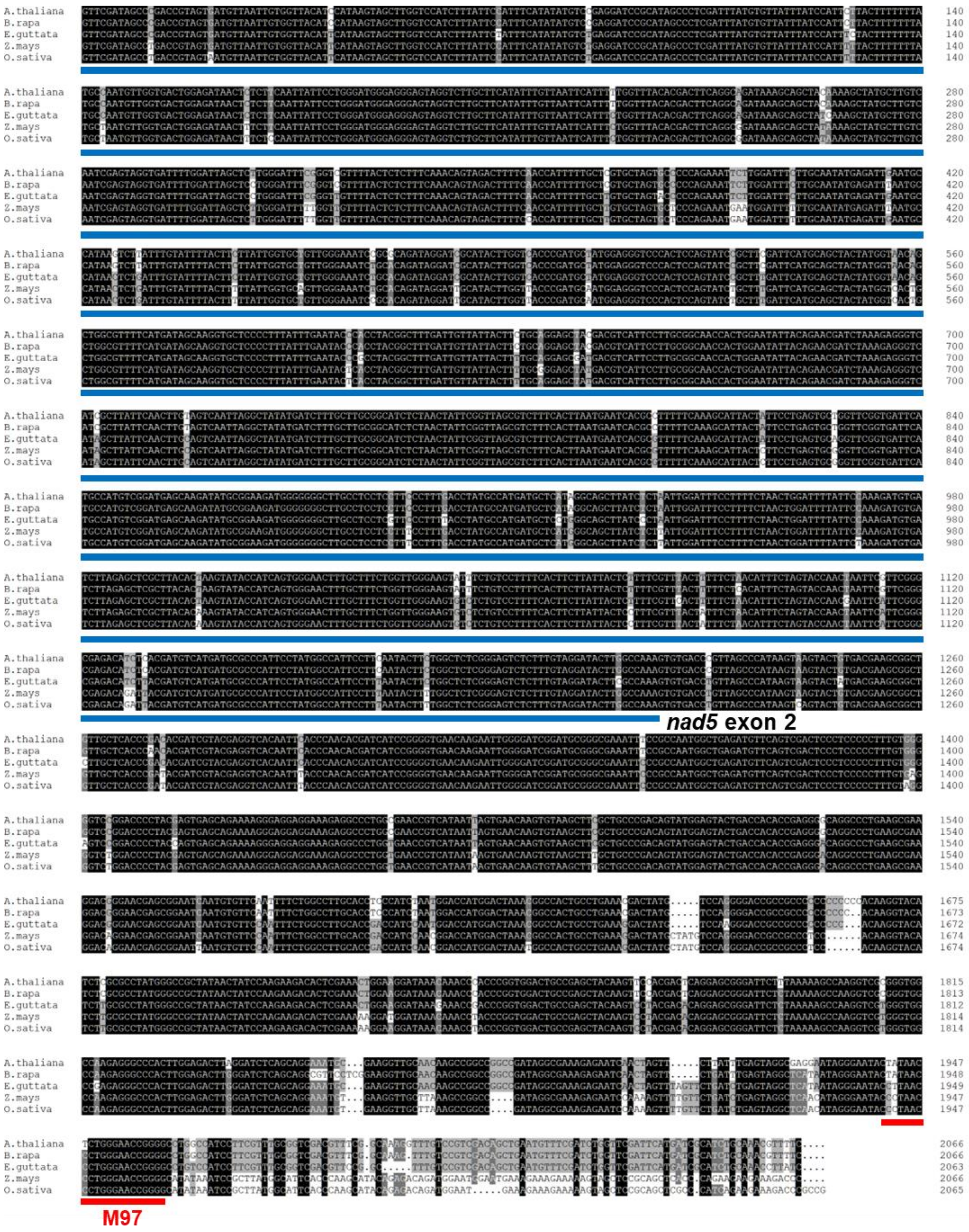

(D)

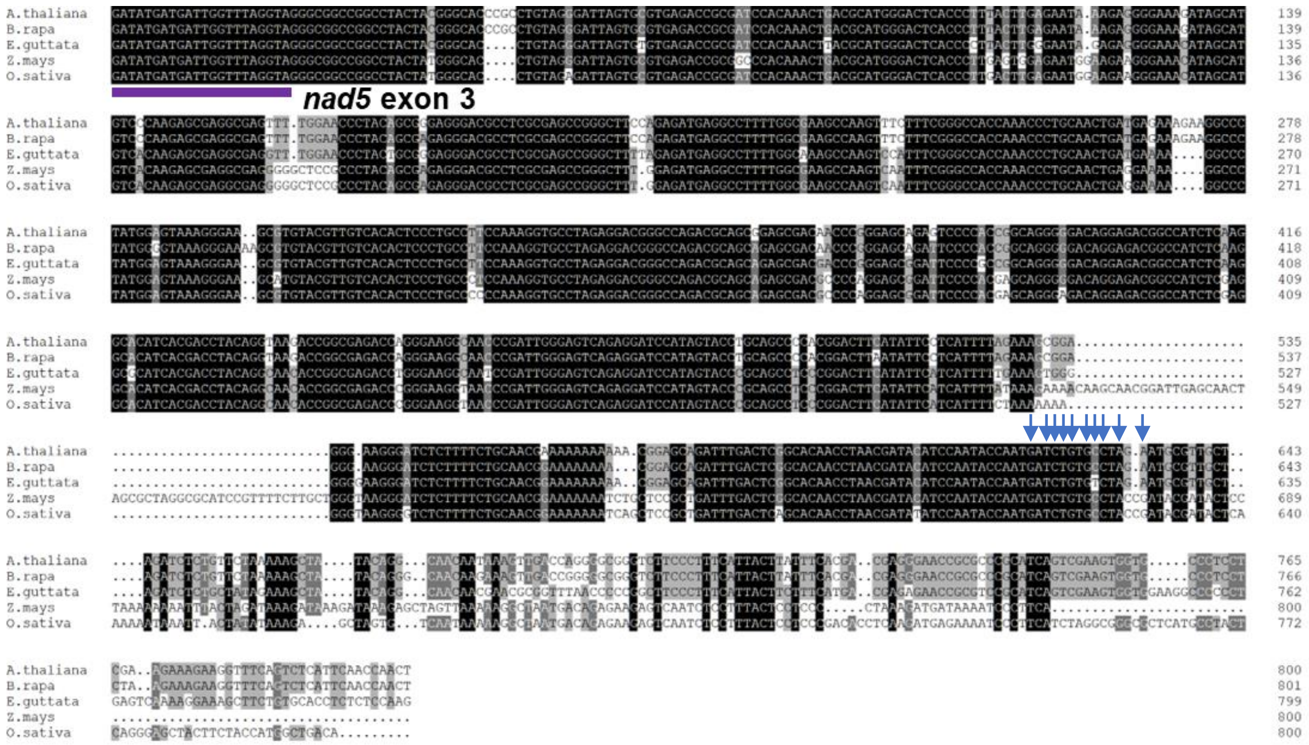

Supplementary Figure S6. Multiple sequence alignment of the 3' regions of *nad1* exon 1 (A), *nad1* exon 2-3 (B), *nad5* exon 1-2 (C) and *nad5* exon 3 (D) precursors from a representative selection of dicot (*Brassica rapa*, *Erythranthe guttata*) and monocot (*Oryza sativa*, *Zea mays*) species. The regions corresponding to the binding sites (BS) of the stabilizing PPR proteins MSP1 and MTSF2 are framed in blue and green, respectively, while the M97 CosRNA identified in Arabidopsis is underlined in red. The different 3' ends mapped for the *nad5* exon 3 precursor are shown as blue arrows.

Supplementary Table S1. List of primers used in this study.

Oligonucleotides	Sequence 5'-3'	Experiment
PPR-P3-4	AATGATTTGGAGGCTTATGCC	Genotyping
PPR-P3-3	TCATCCAGGGTCAAACTGAAC	
LBSALK2	GCTTTCTTCCCTTCCTTTCTC	
GW PPR-P3F	CAAAAAAGCAGGCTATGGTCGTGTTCCCAAAGTC	Complementation; GFP expression
GW PPR-P3R2	CAAGAAAGCTGGGTCGCGATTCACGACAGCTCGAA	
nad2e1	GCAGAATTCGTTCGGATC	mRNA probes for Northern blot
nad2e8	TATGAACTGAGTGCCATTTGA	
nad2 int1-1	TCCTAGAAACGGCGGCAAC	
nad2 int1-2	CTCACCCCATGGCACCTTTT	
nad2 int2a-1	ATGACGAAAGGAGAGTCGGC	
nad2 int2a-2	TTCTGGCCCCTGTGAACATC	
nad2 int2b-1	CCAACGGGGAATAGAAGCGA	
nad2 int2b-2	ATACGGCTCGCACAAAGACT	
QMnad2intron2F	CCCGATCCGATAGTTTACAA	RIP-qPCR
QMnad2exon2R	AATATTTGATCTTAGGTGCATTTTC	
nad2 int2-8	ATCTCCTACGCGCGAAATCA	
nad2 int2-9	GTATCGGGCCCCTTTTCGTT	
nad2 int2-3	TCCCTTCTGGGGTATTCCGA	
nad2 int2-4	ATCCCTTCGGACCTGGAGAA	
nad2 int2-6	CGACCGGATCAGAGTGAAACA	
nad2 int2-7	AACCGTGAGCAGTAAGCAGC	
nad2 int2-10	AGCTTTGGGATTCGCTCACT	
nad2 int2-11	TTCCCTTGTCTGAGAACCGC	
nad2 int2-12	AATGTGGGGAGGAATAGTGCG	
nad2 int2-13	CACTGGTCCTGCTGTGAGG	
nad2 int2-14	ACAGGTTCTAGGTCAAGCGA	
nad2 int2-15	AAAGGGCAGCGGAGGAATAC	
nad2 int2-16	AAGGCGAGCAATCTGTAGGT	
nad2 int2-17	TTGGGAAAGTGAGCCGATCC	
nad2 int2-18	ATCGGCTCACTTTCCCAATC	
nad2 int2-19	ATTGAGCTAACGCCCTCTGT	
nad2 int2b-3	ATGTGCTGTTGTCGCATGAA	
nad2 int2b-4	GAACGGACTCTTTGCCTTGG	
nad2 int2b-5	AAGGGAAAGGGGAGAGTGGT	
nad2 int2b-6	GCCTTCCTACGCTGACGAAT	
nad2 int2b-7	ATTCGTCAGCGTAGGAAGGC	
nad2 int2b-8	ACTATAAGCCAGCCGGAAGC	
nad2 int2b-9	TCCGGCTGGCTTATAGTCCT	
nad2 int2b-10	ATGTATCGGCTTGGCTTCGT	
Primer 1	CGACCGGATCAGAGTGAAACA	PCR for nad2 exon1-2 extension
Primer 2	AAGAAAAAGAAAGAAGAAAGA	
Primer 3	TTTCGACCTATACTATAGTAA	
Nad1M-R	CCAAGTATTTCAGCTGGAAC	cRT-PCR
CRT nad1 intron 4	GACTCAGCAGCAGTGCGGAATTGAGT	
cRTNad1Intron-2	GTCGACCACCACATAAGCCA	
cRTNad1Intron-1	GTTCTTGCCGGGAGAGTAGC	
qnad2-int2-2F	AACGAAAACCACCTCGACCG	
Nad2a exon1 R	TAATCCAAGCCAACCCACAT	
CRT-nad5-ex1	CCGAGCAGGGGCAAAAATACG	
CRT-nad5-int2a	GAGGGCCCACTTGGAGACTT	
CRT-nad5-ex3	CCAATCATCATATCGGTCCCT	
CRT-nad5-int3b-2	CACATCACGACCTACAGGTAAG	

Supplementary Table S2. List of antibodies used in this study.

Antibody	Full Name of detected proteins	Host	Dilution	Source
**Nad7**	NADH-ubiquinone oxidoreductase chain 7	Rabbit	1 : 2,000	(Pineau et al., 2008)
**Nad9**	NADH-ubiquinone oxidoreductase chain 9	Rabbit	1 : 5,000	(Lamattina et al., 1993)
**RISP**	Rieske iron-sulfur protein	Rabbit	1 : 5,000	(Carrie et al., 2010)
**CYT *c***	Cytochrome *c*	Rabbit	1 : 5,000	Agrisera (AS08 343A)
**Cox2**	Cytochrome oxidase subunit II	Rabbit	1 : 1,000	Agrisera (AS04 053A)
**ATPβ**	Beta subunit of ATP synthase	Chicken	1 : 5,000	Agrisera (AS05 085)
**Porin**	The channel-forming protein	Mouse	1 : 500	(Taylor et al., 2003)
**AOX1a**	Alternative oxidase 1A	Rabbit	1 : 1,000	Agrisera (AS04 054)
**CA2**	Gamma-type carbonic anhydrase 2	Mouse	1 : 5,000	(Sunderhaus et al., 2006)
**GFP [3H9]**	Green fluorescent protein epitope	Rat	1 : 1,000	Chromotek (3H9)

Carrie C, Giraud E, Duncan O, Xu L, Wang Y, Huang S, Clifton R, Murcha M, Filipovska A, Rackham O, Vrielink A, Whelan J (2010) Conserved and novel functions for Arabidopsis thaliana MIA40 in assembly of proteins in mitochondria and peroxisomes. J Biol Chem **285:** 36138-36148

Lamattina L, Gonzalez D, Gualberto J, Grienenberger JM (1993) Higher plant mitochondria encode an homologue of the nuclear-encoded 30-kDa subunit of bovine mitochondrial complex I. Eur J Biochem **217:** 831-838

Pineau B, Layoune O, Danon A, De Paepe R (2008) L-galactono-1,4-lactone dehydrogenase is required for the accumulation of plant respiratory complex I. J Biol Chem **283:** 32500-32505

Sunderhaus S, Dudkina NV, Jansch L, Klodmann J, Heinemeyer J, Perales M, Zabaleta E, Boekema EJ, Braun HP (2006) Carbonic anhydrase subunits form a matrix-exposed domain attached to the membrane arm of mitochondrial complex I in plants. J Biol Chem **281:** 6482-6488

Taylor NL, Rudhe C, Hulett JM, Lithgow T, Glaser E, Day DA, Millar AH, Whelan J (2003) Environmental stresses inhibit and stimulate different protein import pathways in plant mitochondria. FEBS Lett **547:** 125-130
